# Dose Up-Titration of a Proprotein Convertase Subtilisin/Kexin Type 9 Inhibitor for Markedly Elevated Lipoprotein(a) in a Patient With Familial Hypercholesterolemia: A Case Report

**DOI:** 10.7759/cureus.105692

**Published:** 2026-03-23

**Authors:** Nobuaki Suzuki, Hisako Kishi

**Affiliations:** 1 Division of Cardiology, Teikyo University Hospital - Mizonokuchi, Kawasaki, JPN; 2 Department of Internal Medicine, Kishi Medical Clinic, Tokyo, JPN

**Keywords:** hyperlipidemia treatment, ldl cholesterol, lipoprotein (a), old myocardial infarction, pcsk-9 inhibitor

## Abstract

Lipoprotein(a) (Lp(a)) is an independent risk factor for atherosclerotic cardiovascular disease. Although proprotein convertase subtilisin/kexin type 9 (PCSK9) inhibitors modestly reduce Lp(a) levels, evidence regarding their dose-dependent effects on patients with extremely high Lp(a) levels is limited.

A 69-year-old woman was diagnosed with dyslipidemia because of high low-density lipoprotein cholesterol (LDL-C) levels (>180 mg/dL) and was initiated on atorvastatin at a dose of 10 mg/day. Two years later, she developed a myocardial infarction, and heterozygous familial hypercholesterolemia was subsequently diagnosed. Eight years later, despite achieving optimal LDL-C levels using high-intensity statin therapy, ezetimibe, and a half-dose of a PCSK9 inhibitor (evolocumab 140 mg monthly), her Lp(a) levels remained high. Up-titration of evolocumab from 140 mg monthly to 140 mg every two weeks resulted in a 34.7% reduction in Lp(a) levels; however, the value only decreased to 82.5 mg/dL.

This case indicates a dose-dependent effect of PCSK9 inhibitor therapy on Lp(a) reduction; however, the magnitude of lowering may be insufficient in patients with extremely high baseline Lp(a) levels.

## Introduction

Lipoprotein(a) (Lp(a)) is an independent and genetically determined risk factor for atherosclerotic cardiovascular disease, exerting proatherogenic, proinflammatory, and prothrombotic effects [1,2]. An Lp(a) ≥50 mg/dL constitutes a risk-enhancing factor [3]. Currently available lipid-lowering agents and lifestyle interventions have minimal impact on lowering Lp(a) [4]. Although statins remain the cornerstone of lipid-lowering therapy, they do not reduce Lp(a) levels. Proprotein convertase subtilisin/kexin type 9 (PCSK9) inhibitors are recommended as adjunctive therapy when low-density lipoprotein cholesterol (LDL-C) targets are not achieved [5], and can modestly lower Lp(a) levels [6]. However, data on their efficacy in patients with extremely high Lp(a) levels, particularly regarding dose up-titration, are scarce. In this report, we present a case illustrating the effect of PCSK9 inhibitor dose escalation on markedly high Lp(a) levels.

## Case presentation

**Figure 1:**
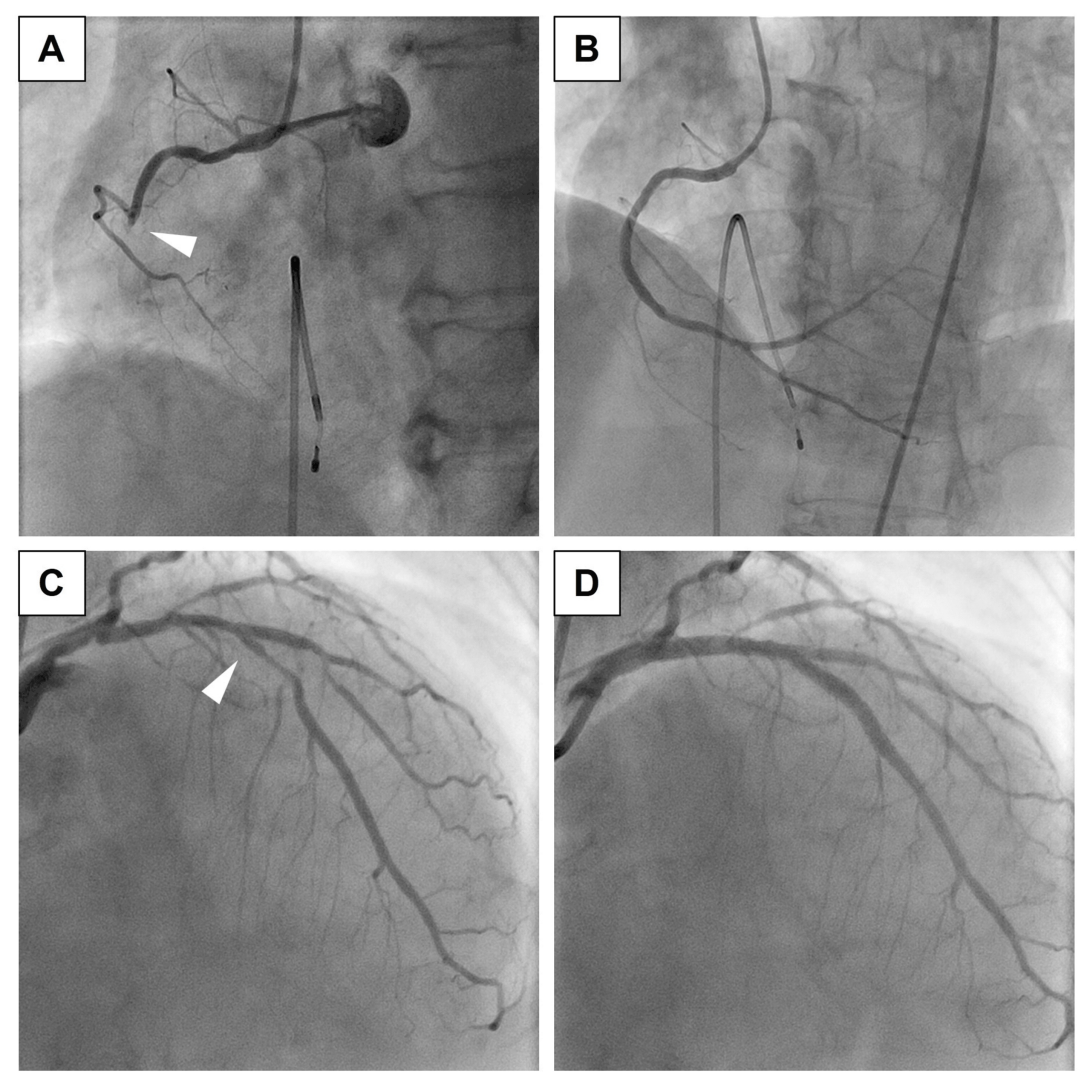
Coronary angiography Emergent angiography revealed total occlusion (white arrow) in the right coronary artery (A). After elective percutaneous coronary intervention, an optimal result was achieved (B). Staged percutaneous coronary intervention was performed for a 90% stenosis (white arrow) in the left anterior descending coronary artery: before (C) and after (D). (A) and (B): Left anterior oblique cranial view; (C) and (D): Right anterior oblique cranial view.

**Figure 2:**
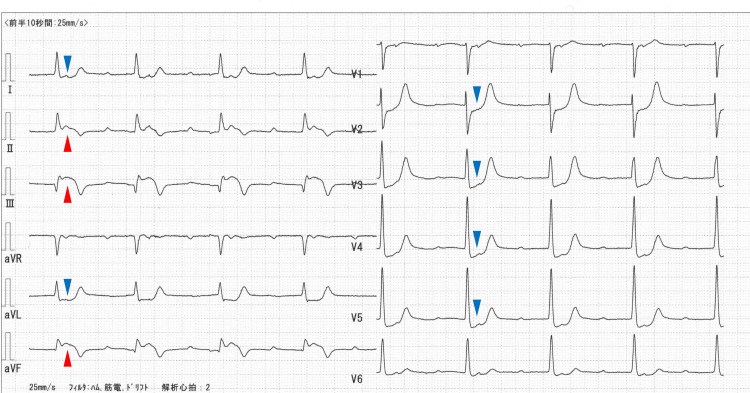
Electrocardiogram On admission, the electrocardiogram revealed ST-segment elevation (red arrows) in leads II, III, and aVF, along with reciprocal changes (blue arrows) in leads I, aVL, and V2-V5. Concomitantly, a complete atrioventricular block was observed.

A 69-year-old woman was diagnosed with dyslipidemia because of high LDL-C levels (>180 mg/dL) and was initiated on atorvastatin at a dose of 10 mg/day. Two years later, she developed an ST-elevation myocardial infarction (Figure 1) and was subjected to primary percutaneous coronary intervention (PCI) with stent implantation (Resolute Integrity; Medtronic, Inc., Minneapolis, MN, USA; 2.75×30 mm, 2.75×26 mm, and 3.0×9 mm) in the right coronary artery (Figures 2A-2B). The maximum creatinine kinase level was 888 IU/L. Subsequently, staged PCI with stent implantation (Nobori; Terumo, Tokyo, Japan; 3.5×28 mm and 3.0×28 mm) was performed to treat a 90% stenosis in the left anterior descending coronary artery (Figures 2C-2D).

Despite treatment with atorvastatin 20 mg/day and ezetimibe 10 mg/day, LDL-C levels remained high at 148 mg/dL; thus, a PCSK9 inhibitor was initiated. Heterozygous familial hypercholesterolemia was diagnosed based on the presence of Achilles tendon xanthomas, measuring >8 mm on X-ray imaging. The initiation of PCSK9 inhibitors was considered; however, owing to economic constraints, therapy was commenced at half the standard dose. Despite half-dose administration (alirocumab 75 mg/month for six years, followed by evolocumab 140 mg/month), LDL-C levels were consistently maintained at <70 mg/dL, in accordance with guideline-recommended targets [7]. Other than coronary artery disease, she had a history of rheumatoid arthritis and osteoporosis. She had no family history of atherosclerotic cardiovascular disease.

**Figure 3:**
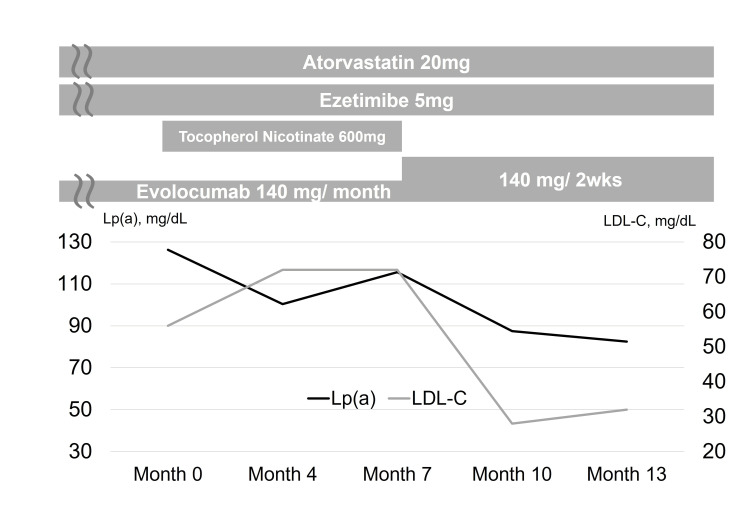
Clinical course Up-titration of evolocumab modestly reduced Lp(a) levels. Lp(a): lipoprotein(a); LDL-C: low-density lipoprotein cholesterol

Eight years after the initial myocardial infarction, Lp(a) levels were measured according to guideline recommendations [8] and were found to be markedly high at 126.3 mg/dL (immunoturbidimetric assay). Therefore, additional medication (tocopherol nicotinate 600 mg/day; up-titration of evolocumab) was planned. First, tocopherol nicotinate 600 mg/day was administered (Figure 3 and Table 1), leading to a modest reduction in Lp(a) to 115.6 mg/dL (8.5% decrease). Whereas up-titration of evolocumab from 140 mg monthly to 140 mg every two weeks reduced Lp(a) to 82.5 mg/dL (34.7% decrease), LDL-C markedly decreased from 80 mg/dL to 28 mg/dL (65.0% decrease). The patient’s clinical course and laboratory data are summarized in Figure 3 and Table 1. For the measurement of LDL-C and Lp(a), all blood samples were obtained in the fasting state and, where applicable, immediately prior to the administration of a PCSK9 inhibitor.

**Table 1:** Laboratory data Hb: hemoglobin; FBS: fasting blood sugar; T-bil: total bilirubin; GGT: gamma-glutamyltransferase; CK: creatine kinase; Cr: creatinine; UA: uric acid; HDL-C: high-density lipoprotein cholesterol; LDL-C: low-density lipoprotein cholesterol; TG: triglyceride; Lp(a): lipoprotein(a)

Tests	Results					Units	Reference Range
	Month 0	Month 4	Month 7	Month 10	Month 13		
Hb	11.9	11.2	11.8	12.0	11.5	g/dL	12.0-16.0
FBS	89	82	86	98	85	mg/dL	70-109
T-bil	0.4	0.4	0.5	0.6	0.4	mg/dL	0.2-1.1
GGT	44	47	34	40	43	IU/L	0-45
CK	51	54	59	62	40	IU/L	40-160
Cr	0.93	0.93	0.86	0.88	0.8	mg/dL	0.31-0.88
UA	5.5	5.6	5.0	5.1	4.4	mg/dL	2.4-7.0
HDL-C	67	75	68	82	77	mg/dL	40-100
LDL-C	56	72	80	28	32	mg/dL	70-139
TG	190	177	223	97	176	mg/dL	50-149
Lp(a)	126.3	100.3	115.6	87.4	82.5	mg/dL	0-30

## Discussion

Lp(a) levels are largely genetically determined and remain stable throughout life [9,10]. Murase et al. reported that subjects with markedly elevated serum Lp(a) concentrations (i.e., ≥100 mg/dL) are rarely encountered, but constitute a significant risk factor for ischemic heart disease [11]. They also noted that only a limited number of studies have described the presence of individuals with Lp(a) levels ≥100 mg/dL [12,13].

A study indicated reduced Lp(a) levels in patients treated with tocopherol nicotinate [14]. However, in the present case, the administration of tocopherol nicotinate only led to minimal Lp(a) reduction. While previous studies have demonstrated modest reductions in Lp(a) levels with PCSK9 inhibitors [6,15], evidence regarding the effect of dose up-titration has been limited. Although the up-titration of the PCSK9 inhibitor dose substantially reduced Lp(a) levels in the present case, the achieved Lp(a) level remained well above proposed thresholds associated with increased cardiovascular risk [16]. This underscores the limitations of current therapies in patients with extremely high Lp(a) levels. Further re-examination across multiple cases, under standard dosing conditions, is warranted. Intra-individual variability in Lp(a) may have influenced the data of this case; however, the previous report showed that the variability is limited when the Lp(a) levels are extremely high [17].

Emerging therapies targeting Lp(a), such as antisense oligonucleotides and small interfering RNA agents, have led to marked reductions in Lp(a) levels [18-20]. If these therapies can improve cardiovascular outcomes, they may be a potential option for patients with refractory Lp(a) elevation.

## Conclusions

In this case, the dose up-titration of the PCSK9 inhibitor modestly reduced the Lp(a) level, but was too high to reduce the cardiovascular risk. In patients with extremely high Lp(a) levels, the magnitude of reduction achieved with current therapies may be insufficient. Novel Lp(a)-targeted therapies may be required to achieve clinically meaningful risk reduction in such patients.
